# Novel genetic variants data for adaptation to hypoxia in native chickens

**DOI:** 10.1186/s13104-023-06493-x

**Published:** 2023-09-21

**Authors:** Atieh Moradi, Hamed Kharrati-Koopaee, Morteza Fardi, Mehdi Farahmandzadeh, Fatemeh Nowroozi

**Affiliations:** 1 School of Biological Science, The University of Hong Kong, Hong Kong, China; 2 Institute of Biotechnology, Shiraz University, Shiraz, Iran; 3 North Region Branch, Agricultural Biotechnology Research Institute of Iran (ABRII), Agricultural Research, Education and Extension Organization (AREEO), Rasht, Iran

**Keywords:** Native chicken, Hypoxia, Genetic variants

## Abstract

**Objective:**

The genomic response and the role of genetic variants in hypoxia condition are always interesting issues about adaption pathways at genomic level. Herein, we carried out a comparative genomic study between highland and lowland native chickens, in order to identify the adaptive variants in hypoxia condition. We generated more than 20 million genetic variants in highland and lowland chickens. Finally, 3877 SNVs including the mtDNA ones, were discovered as novel adaptive genetic variants. The generated data set can provide new insight about mechanism of adaptation to hypoxia at genomic level.

**Data description:**

To investigate the role of genetic variants in adaptation to hypoxia, 10 whole-genome sequencing data sets associated to highland and lowland native chickens were provided. DNA was extracted by salting-out protocol. Paired-end 125 bp short reads were sequenced by Illumina Hiseq 2000. Variants calling of highland and lowland native chickens were performed by fix ploidy algorithm in CLC Genomic Workbench. Total genetic variants of highland chickens were compared to lowland chickens in order to identify the differential genetic variants (DGVs) between highland and lowland chickens. In this way, 3877 novel SNVs (VCF format) including the mtDNA ones, were deposited at EBI database (https://identifiers.org/ena.embl:ERZ491574) for the first time.

## Objective

Chicken (*Gallus gallus demesticus*) is one of the widespread domestic bird in the world. Origin of chicken’s domestication has always been a challenge. However, the outcomes of investigations showed chickens have been domesticated more than 10,000 years ago from Red Jungle Flow subspecies *Gallus gallus spadiceus* that is distributed in northern Thailand, China, and Myanmar [1]. Food production, religions ideas and cock fighting are the most important reasons for the beginning of chicken domestications [2]. It is believed, genome is dynamic and it responses to environmental conditions. Alterations of genetic variants as genome response play a critical role in domestication and adaptation of chickens [3]. Altitude is distance above sea level and areas that are above 2,400 m of sea level are usually considered as high-altitude condition. Hypoxia is the most important risk factor in high-altitude areas that organisms must adapt to this harsh environment in order to increase the probability of viability [4]. Hypoxia means low oxygen and adaptation to hypoxia is a complex process that includes some biological pathways and gene networks [5]. Therefore, understanding the genetics factors associated with adaptation to high-altitude conditions in domestic animals provide new science for finding the adaptation process [6].

There are studies carried out in order to identify the genetic factors related to high-altitude conditions. Results of these studies show that hypoxia is involved in cerebral edema, tumorigenesis, myocardial ischemia and some genes including Endothelin 1 (*EDN1*), Erythropoietin (*EPO*) and Aldosterone synthase (*CYP11B2*) are reported as the key genes of hypoxia adaptation [7–9]. Investigations indicated that highland chickens are adapted based on factors including the small body size, the ability to outstand foraging, high hatchability, large organs (liver, heart and lungs), and higher hemoglobin concentration [8].

Due to different climates and altitudes, there is considerable genetic diversity among indigenous chickens in Iran [10]. Historical evidence shows that Iran is one of the oldest poultry breeding centers in the world. Chicken have been kept since many years ago by farmers and consequently they have adapted to local environment conditions by genetic changes [11]. Previously, we performed a comparative genomic study by whole genome sequencing data between highland and lowland native chickens in order to identify the adaptive genetic variants in hypoxia condition [5].

Our founding indicated that adaptive variants are involved in DNA repair, organs development, immune response and histone binding. Cellular component analysis of variants showed that mitochondrion is the most important organelle for hypoxia adaptation. High-altitude associated with variant discovery highlighted the importance of *COX3*, a gene involved in cell respiration, in hypoxia adaptation [5]. Finally, 3877 novel SNVs including the mtDNA ones, were submitted to EBI (PRJEB24944). The submitted genetic variants of native chickens provided new insights about adaptation mechanisms and highlights the importance of valuable genomic variants in chickens.

## Data description

The current data set and additional methods details were published previously in Scientific Report journal [5]. In order to identify adaptive genetic variants five blood samples were collected from Mazandraran province (Altitude = 54 m) as lowland chickens and five blood samples were obtained from Isfahan province (Altitude = 2087 m) as highland chickens. DNA was extracted by salting-out protocol. Nanodrop (ratio 260/280 (nm)) and agarose gel (1%) electrophoresis was applied to quantity and quality controls of the provided DNA [12]. Paired-end 125 bp short reads were sequenced by Illumina Hiseq 2000 [1]. Around 0.94 Gbp and 22.1 Gb data were provided [5]. Reference genome and annotations were downloaded from the Ensembl database (fp://fp.ensembl.org/pub/relase-84/fasta/gallus_gallus). CLC Genomics Workbench (version:8.5.1) [13] was applied to adaptors trimming, quality control and mapping short reads against reference genome. Variants calling of highland and lowland native chickens were carried out by fix ploidy algorithm in CLC Genomic Workbench (version:8.5) under default parameters and more than 20 million genetic variants were generated. Total genetic variants of highland chickens were compared to lowland chickens in order to identify the differential genetic variants (DGVs). In this way, a total 114,634 DGVs was reported between highland and lowland chickens. Known variants annotation was utilized to identify the novel genetic variants that have not been reported previously in database of single nucleotide polymorphisms dbSNPs (www.ncbi.nlm.nih.gov/snp). Finally, 3877 novel SNVs (VCF format) were submitted to EBI database (https://www.ebi.ac.uk/). The submitted data is available in the following link (https://identifiers.org/ena.embl:ERZ491574) [14]. Table 1 shows the details of submitted data and direct download link. Variants discovery projects produce numerous variations. Thereby, validating the variants is highly required. Here, validations were carried out for two groups of detected variants. First, novel differential SNVs between highland and lowland chickens, and second, mtDNA variations in highland chickens. The whole genome sequencing and genetic variants calling of other five whole genomes of highland samples (Isfahan) was carried out, separately. Finally, in order to validate the reported novel genetic variants, each novel SNV’s regions and chromosomes were evaluated in the new five samples by R program (https://www.r-project.org) [5].”

**Table 1:** The novel genetic variants data sets for adaptation to hypoxia in native chickens at ENA (European Nucleotide Archive) database

Label	Name of data file	File types	Data repository and identifier
Data file 1	Novel-mt-DNA-lowland-male	VCF(vfc.gz)	European Nucleotide Archive https://identifiers.org/ena.embl:ERZ491574 [14]
Data file 2	Novel-mt-DNA-lowland-female	VCF(vfc.gz)	European Nucleotide Archive [14]
Data set 3	Novel-mt-DNA-highland-male	VCF(vfc.gz)	European Nucleotide Archive [14]
Data file 4	Novel-mt-DNA-highland-female	VCF(vfc.gz)	European Nucleotide Archive [14]
Data file 5	Novel-differential-variants-male-somatic-chr	VCF(vfc.gz)	European Nucleotide Archive [14]
Data file 6	Novel-differential-variants-male-chr-z	VCF(vfc.gz)	European Nucleotide Archive [14]
Data file 7	Novel-differential- variants-female-somatic-chr	VCF(vfc.gz)	European Nucleotide Archive [14]
Data file 8	Novel-differential-variants-female-chr-Z	VCF(vfc.gz)	European Nucleotide Archive [14]

### Limitations

In the current investigation, 10 native chickens were studied to describe the adaptive variants in hypoxia condition. Therefore, the generated genetic variants could not present a comprehensive information of adaptation process in native chickens.

## Data Availability

The genetic variant data described herein have been deposited in EBI database (European Nucleotide Variation) as novel genetic variants in (VCF) format (https://identifiers.org/ena.embl:ERZ491574) under the accession number of PRJNA532674.

## Abbreviations

Gb: Giga byte
Gbp: Giga base pair
DNA: Deoxyribonucleic acid
SNP: single nucleotide polymorphism
SNV: single nucleotide variation
DGVs: differential genetic variants
VCF: variant call format
